# Abundance of Gut Microbiota, Concentration of Short-Chain Fatty Acids, and Inflammatory Markers Associated with Elevated Body Fat, Overweight, and Obesity in Female Adolescents

**DOI:** 10.1155/2019/7346863

**Published:** 2019-12-17

**Authors:** Valter Paulo Neves Miranda, Paulo Roberto dos Santos Amorim, Ronaldo Rocha Bastos, Eliane Rodrigues de Faria, Maria Eliza de Castro Moreira, Sylvia do Carmo Castro Franceschini, Maria do Carmo Gouveia Peluzio, Célia Lucia de Luces Fortes Ferreira, Silvia Eloiza Priore

**Affiliations:** ^1^Department of Nutrition and Health and Department of Physical Education, Universidade Federal de Viçosa, Minas Gerais CEP 36570-900, Brazil; ^2^Department of Physical Education, Universidade Federal de Viçosa, Viçosa, Minas Gerais CEP 36570-900, Brazil; ^3^Department of Statistics-ICE, Universidade Federal de Juiz de Fora, Juiz de Fora MG, Brazil CEP 36036-330; ^4^Department of Nutrition, Universidade Federal de Juiz de Fora, Juiz de Fora, MG, Brazil CEP 36036-900; ^5^Department of Nutrition and Health, Universidade Federal de Viçosa, Minas Gerais CEP 36570-900, Brazil; ^6^Department of Food Technology, Universidade Federal de Viçosa, Minas Gerais CEP 36570-900, Brazil

## Abstract

**Background and Aims:**

Overweight is ever more prevalent in the pediatric population, and this cardiometabolic factor can be associated with inflammatory markers, gut microbiota composition, and short-chain fatty acid (SCFA) concentrations. The aim of this study is to evaluate to what extent the abundance of gut microbiota phyla, SCFA concentrations, and inflammatory markers are associated with elevated body fat percentage (BF%), overweight, and obesity in female adolescents.

**Methods:**

An experimental and comparative study was conducted with 96 girls 14 to 19 years old. They were divided into 3 groups: G1—eutrophic (EUT) and adequate BF%; G2—EUT and high BF%; and G3—overweight (OW) or obese (OB) and high BF%. Waist circumference (WC), waist to height ratio (WtHR), and neck circumference (NC) were analyzed as indicators of central visceral adiposity. The BF% was evaluated by DEXA equipment. A food frequency questionnaire was used to evaluate the main types of food consumed in a week. The abundance of the Firmicutes, Bacteroidetes, and Proteobacteria phyla was measured by real-time polymerase chain reaction (RT-qPCR), and the SFCA concentrations (acetic, butyric, and propionic) were determined by high-performance liquid chromatography (HPLC). The inflammatory markers leptin, tumor necrosis factor-alpha, interleukin-6, and high-sensitivity C-reactive protein (hs-CRP) were assessed.

**Results:**

Female adolescents in groups G2 and G3 had greater central visceral adiposity and leptin concentration than those in group G1. No association was found between gut microbiota phyla abundance and SFCA concentrations in any of the groups. WC and frequency of consumption of oily and fatty foods were associated with Firmicutes abundance and SFCA concentrations. Girls with high WC also had the greatest leptin (*p* < 0.001) and hs-CRP (*p* = 0.035) concentrations.

**Conclusions:**

Inflammatory markers showed association with increased BMI and high BF% in female adolescents. The abundance of Firmicutes was associated with WC and NC, but not with BMI classification or BF%. Specifically, WC and the consumption of oils and fats showed correlation with SCFA concentrations. Different anthropometric indicators, such as NC and WC, should be incorporated into the clinical evaluation of the nutritional status of individuals in the adolescent population.

## 1. Introduction

Overweight and obesity cases have increased considerably in recent years among adolescents, to the point that both can be currently considered major public health problems [1]. Environmental, dietary, and behavioral patterns contribute to excessive accumulation of body fat [1]. In Brazil, epidemiological data have shown that girls are more physically inactive and sedentary and consume more fried foods, sweets, and cookies than boys [2].

In addition to being a risk factor for cardiometabolic diseases, overweight and obesity can be directly related to changes in the composition of the gut microbiota and its metabolites [3]. One of the possible mechanisms underlying the interplay between microbiota and host metabolism is through appetite-regulating hormones, including leptin, ghrelin, and glucagon-like peptide-1 [4].

The human gut microbiota is a complex community of 100 trillion archaeal and bacterial cells distributed over more than 1,000 species [5]. In a healthy individual, almost 90% of bacteria phyla are Firmicutes and Bacteroides [6]. Each person has a distinct and highly variable microbiota, but a conserved set of gut colonizers (the core gut microbiota) and genes (the core microbiome) is shared among individuals [5] and may be required for the correct functioning of the gut [6].

Dysbiosis is the imbalance between colonizing bacteria of the gastrointestinal tract (GIT). Gut microbiota composition is altered in people who are obese, and it can respond to changes in body weight [6]. An elevated Firmicutes-to-Bacteroidetes ratio has been observed in the obese study population [7–9] Proteobacteria, Actinobacteria, and Verrucomicrobia are other phyla in smaller amounts in the human GIT. Specifically, Actinobacteria have been found in smaller amounts in overweight and obese adolescents than in lean adolescents [7]. The imbalance between these phyla can influence the immune system and the energy storage regulation of the host.

Enzymes produced by the intestinal microbiota, mainly by species of the Firmicutes phylum, ferment polysaccharides that are not digested in the GIT and produce acetic, butyric, and propionic acids [10]. These short-chain fatty acids (SCFA) are the final product of bacterial fermentation of nondigestible carbohydrates, and they can alter the body's energy usage and metabolic profile through enteroendocrine cell signaling, adipogenesis, and production of insulin-like growth factor-1 [3]. SCFAs are volatile organic fatty acids that contribute up to 10% of an individual's basal energy expenditure, being the main energy source for cells in the intestinal wall [10].

Furthermore, the SCFAs are involved in lipid and glucose metabolism, which to some extent confirms their influence in the manifestation of risk factors for metabolic disease [11]. A higher concentration of SCFAs in feces can be related to excess body fat in humans [11].

The activity of GIT bacteria in overweight or obese people remains controversial in the literature [4]. Recent scientific evidence has shown the imbalance of intestinal microbiota in children and adolescents to be related to the increase in obesity cases in adults [7]. However, few studies have evaluated the relationship between body composition and food frequency with the GIT bacterial phyla and their metabolites in the pediatric population [4, 7].

Thus, the aim of this study was to evaluate to what extent the abundance of gut microbiota phyla, SCFA concentrations, and inflammatory markers are associated with elevated body fat, overweight, and obesity in female adolescents.

## 2. Materials and Methods

An experimental and comparative study was carried out with female adolescents ranging from 14 to 19 years of age, enrolled at public schools in Viçosa, MG, Brazil, and living in the same city. All participants were part of an earlier research project called “Evaluation of Lifestyle of Female Adolescents through Latent Class Analysis Approach,” which is described elsewhere [12]. The girls who participated in the study were randomly selected and grouped into three different groups according to their body mass index (BMI) classification and body fat percentage (BF%).

First, 35 female adolescents were randomly selected to make up each of the three groups: group 1 (G1—control group), eutrophic (EUT) and adequate BF%; group 2 (G2), EUT and high BF%; and group 3 (G3), high BF% and either overweight (OW) or obesity (OB).

The following inclusion criteria were adopted: being between 14 and 19 years old, having started menstrual function (menarche), voluntarily accepting to participate in the project (or having signed permission from the parents or legal guardian, if under 18), having no previous diagnosis of any type of chronic or infectious disease, not being in use of any type of antibiotic or other types of medicine that interferes with metabolism, not participating in other research involving body composition assessment or nutritional status control, not being in use of probiotic or prebiotic supplements, and having taken no antibiotics for the past three months.

## 3. Ethical Considerations

The study was approved by the Committee for Ethics in Research with Human Beings of the Universidade Federal de Viçosa (UFV) and filed on the Brazil Platform under the reference number 30752114.0.0000.5153 (decision 700.976/2014). The present project followed the rules set forth by the Brazilian National Health Council Resolution 466/12. Each volunteer only took part in the project after turning in the Assent Form and the Informed Consent Form, signed, respectively, by themselves and by their parents or legal guardians. All experimental procedures were in accordance with the ethical standards of the institutional committee for research with human beings and with the 1964 Helsinki declaration and its later amendments or comparable ethical standards.

## 4. Data Collection Procedures

Data collection procedures started in June 2014 and finished in December 2015. The first stage took place in the schools after consulting with and getting approval from the director. The students received an explanation about the procedures and were given the Assent and Informed Consent forms to be properly signed and handed back. Both contained detailed descriptions of the project and assured the safety, confidentiality, and privacy of the collected information.

The second stage of the experiments occurred in the Health Division (HD) of the Universidade Federal de Viçosa (UFV). In this stage, all the body composition measurements, biochemical tests, and stool sample collections by the adolescents were carried out to analyze the microbiota and short-chain fatty acids.

## 5. Body Composition Assessment

A previously trained female researcher performed all the anthropometric measurements. Weight was measured on an electronic digital scale (Kratos®, Campinas, SP, Brazil), and height was measured with a portable stadiometer (Alturexata®, Belo Horizonte, Brazil). Subsequently, the body mass index (BMI) was calculated by the *Z*-score in the WHO AnthroPlus software. The BMI classification was based on the cutoff points proposed by De Onis et al. [13].

The participants went through a 12-hour fasting. Total BF% was evaluated by a Dual-Energy X-ray Absorptiometry (DEXA) device (Lunar Prodigy Advance DEXA System analysis version 13.31, GE Healthcare, Madison, WI, USA). The BF% was assessed according to the cutoff points proposed by Williams et al. [14]. A BF% above 30.0% was considered high.

To measure the waist circumference (WC), we used a 2-meter, flexible, and inelastic measuring tape (Cardiomed®, São Luis, MA, Brazil), divided into centimeters and millimeters. Measurements started at the midpoint between the lower margin of the last rib and the iliac crest, on the horizontal plane. For WC classification, the 90th percentile (90^th^ P) was considered the standard [15]. The waist to height ratio (WtHR) was obtained by dividing the waist circumference (cm) by the height (cm).

The neck circumference (NC) was measured at the midpoint of the neck height. The cutoff point used for NC classification was 34.1 cm as observed by Silva et al. [16] in Brazilian adolescents. This value has presented a better balance between sensitivity and specificity to metabolic syndrome risk factors in pubertal adolescents [16].

## 6. Food Frequency Questionnaire (FFQ)

Food frequency was assessed using a simplified version of the food frequency questionnaire (FFQ), which only considered the number of times a week certain food types were consumed. FFQ information was analyzed in terms of the number of days in a week each food type was consumed at least once.

For each food type, the girls were classified as showing either adequate or inadequate food frequency, taking the 75^th^ percentile (*P*_75_) of the whole sample as the cutoff. Specifically, “fruits” (*P*_75_ = 6), “vegetables” (*P*_75_ = 7), “tubers” (*P*_75_ = 4), “dairy products” (*P*_75_ = 7), and “cereals, bread, and pasta” (*P*_75_ = 7) were considered inadequate when less than the 75^th^P, and “sugars and sweets” (*P*_75_ = 7), “oils and fats” (*P*_75_ = 7), and “condiments” (*P*_75_ = 7) were considered inadequate when equal to or greater than the 75^th^P.

The daily number of meals was computed from the responses to breakfast, snack, lunch, afternoon tea, dinner, and supper. The mean value for all seven days was calculated and subsequently categorized relative to the 50th percentile (*P*_50_ = 4.0). Values equal to or less than *P*_50_ were considered a low number of meals.

## 7. Risk Factors for Cardiometabolic Diseases

### 7.1. Biochemical Markers

The biochemical analyses were performed between 07:00 and 09:00 A.M. in the Clinical Analysis Laboratory of the HD from UFV. Blood samples were collected after a 12-hour fast from an antecubital vein and centrifuged at 2225 × *g* for 15 minutes at room temperature (2–3 Sigma, Sigma Laborzentrifugen, Osterode am Harz, Germany).

First, total cholesterol (TC), high-density lipoprotein (HDL), low-density lipoprotein (LDL), very low-density lipoprotein (VLDL), and triglyceride concentrations were analyzed. These analyses were done on blood serum after the material was centrifuged in an Excelsa centrifuge model 206 BL for 10 minutes at 3,500 × *g*. The enzymatic colorimetric method was used to measure TC, HDL, and triglycerides with automation by Cobas Mira Plus equipment (Roche Corp.). LDL was calculated indirectly by the Friedwald formula for triglyceride values lower than 400 mg/dL.

The lipid profile was assessed according to the 2017 Brazilian Guidelines for Dyslipidemia and Prevention of Atherosclerosis [17], with TC, triglycerides, and LDL values considered high when greater than or equal to 150 mg/dL, 100 mg/dL, and 100 mg/dL, respectively. HDL was considered low when the values were less than or equal to 45 mg/dL.

Fasting glycemia was measured by the enzymatic method of glucose oxidase using the Cobas Mira Plus automation device (Roche Corp.) [18].

Fasting insulin was measured by the electrochemiluminescence method and classified according to the Guidelines of the Brazilian Diabetes Society, which considers high fasting plasma insulin higher than 15 *μ*U/mL [18].

The mathematical model Homeostasis Model Assessment-Insulin Resistance (HOMA-IR) was used to calculate insulin resistance using insulin and fasting blood glucose measurements: HOMA‐IR = [(fasting insulin (*μ*U/mL) × fasting blood glucose [mmol/L])/22.5] [16]. Values of HOMA-IR higher than 3.16 were considered elevated [19].

### 7.2. Inflammatory Markers

The evaluated inflammatory markers were as follows: interleukin-6 (IL-6), tumor necrosis factor-alpha (TNF-*α*), leptin, and high-sensitivity C-reactive protein (hs-CRP). For this, 500 *μ*L of serum was separated from each blood sample and stored in an ultrafreezer at -80°C until the day of evaluation. These markers were dosed by the multiplex system Luminex™ xMAP technology (multianalyte profile, x = cytokines) using the HMHEMAG-34K kit (IL-6, TNF-*α*, and leptin).

The MILLIPLEX™ kits were purchased from Merck Millipore Corporation (Merck KGaA, Darmstadt, Germany), and the analyses were performed at the Specialized Laboratory in Clinical Analyses (LEAC-Lab Ltda, São Paulo, SP, Brazil). The acute-phase hs-CRP protein was measured by the immunoturbidimetry method.

### 7.3. Gut Microbiota Analysis

After receiving the feces pots from the patients treated at the (UFV) Health Division, the samples were immediately frozen and stored at -20°C in the Nutrition Biochemistry Laboratory of the Department of Nutrition and Health (UFV) until DNA extraction. This procedure was performed with 200 mg (±20) of feces in a 2 mL microtube; the QIAamp Fast DNA Stool Mini Kit (Qiagen, Hilden, Germany) was used for the extraction, following the manufacturer's instructions. The process of DNA extraction for gut microbiota analysis took place in the Experimental Nutrition Laboratory of the Nutrition and Health Department of the UFV. The DNA samples were subsequently stored at -80°C in the same laboratory.

The real-time polymerase chain reaction technique (RT-qPCR) was used to analyze the microbiota. DNA concentration was determined by absorbance at 260 nm (A260), and the estimated purity was determined by the A260/A280 ratio in a Multiskan™ 1500 spectrophotometer (Thermo Fisher Scientifics; Waltham, MA, EUA). Specific primers were used for different bacterial phyla that characterize the fecal microbiota under quantification (Table 1). The RT-qPCR analyses were performed on a CFX96 Touch™ detection system (Bio-Rad, Berkeley, California) (Primer Express software) using the QuantiNova™ SYBR® Green PCR Kit (Qiagen) detection kit.

All samples were analyzed in duplicate, with each well of the RT-qPCR plate containing 2 *μ*L sample or standard, 300 nM of sense and antisense primers (Alpha DNA), and nuclease-free water for a total of 25 *μ*L. The thermal conditions for the PCR cycle were as follows: initial denaturation of the DNA at 95°C for 10 minutes, followed by 40 cycles of denaturation at 95°C for 10 seconds, annealing of the primer at the optimum temperature for 20 seconds, and extension at 72°C for 15 seconds. A melting curve analysis after the amplification step was performed to ensure the quality and specificity of the RT-qPCR.

Bacteria phyla abundances from each fecal sample were calculated by comparing the Ct values obtained through standard curves from the Primer Express software®. The analysis of the melting curve was done after the amplification of the analyzed bacterial phyla to distinguish the different products of the probably amplified genes. Standard curves were constructed for each experiment using five-fold serial dilutions of bacterial genomic DNA (known concentrations) from pure cultures with 16S rRNA gene ranging from 20 ng to 0.032 ng.

Strains specific to each phylum were used to analyze the abundance of bacteria: Firmicutes, strains from the Tropical Cultures Collection (*Lactobacillus delbrueckii* UFV H2b20 CCT 3744) and Bacteroidetes and Proteobacteria, strains obtained from the American Type Culture Collection (ATCC) (*Bacteroides ovatus* ATCC 8483 and *Escherichia coli* ATCC 11775). The annealing temperatures of *Lactobacillus delbrueckii*, *Bacteroides ovatus*, and *Escherichia coli* were 83.5°C, 82.0°C, and 83.5°C, respectively.

Concentrations of DNA dilutions were assessed after isolation from the pure cultures with the Ct values of the bacteria phyla to verify the accuracy of the RT-qPCR method. The amplification and *R*^2^ efficiencies of equations for each plate analyzed ranged from 1.74 to 2.3 and 0.88 to 0.95, respectively. Efficiency values close to 2 and *R*^2^ of the equation close to 1 confirm the accuracy of the method, according to Stevenson and Weimer [23].

### 7.4. Short-Chain Fatty Acid Analysis

High-performance liquid chromatography (HPLC-UV) was performed for the quantification of acetic, propionic, and butyric acids [24], in the Clinical Analysis Laboratory of the Department of Nutrition and Health of the Federal University of Viçosa. SCFA extraction was performed according to the protocol proposed by Zhao et al. [25], adapted for the analysis of organic acids in HPLC. This process was performed in the Nutrition Biochemistry Laboratory of the Nutrition and Health Department of the UFV.

Initially, stool samples were taken from the freezer and left at room temperature for 30 minutes. Then 5 mL ultrapure water was added to 500 mg of each sample (1 in 1 mL) vortexing each time the water was added, transferring the contents to a Falcon tube of 15 mL, until the microcentrifuge tubes of 2.0 mL of capacity were well cleaned, and all contents were transferred into the Falcon tube. Within this tube, the medium pH was acidified from 2.3 to 3 using HPLC-grade orthophosphoric acid (H3PO4) at a concentration of 12.0%. The pH was checked in the fecal specific meter in the Vitamin Evaluation Laboratory of the UFV.

The Falcon tubes were centrifuged under 4°C refrigeration for 20 minutes at 4,100 rpm, and the supernatant was then transferred to a pair of 2.0 mL microcentrifuge tubes. The microcentrifuge tubes with the filtered supernatant were centrifuged again at 13,500 rpm under refrigeration (4°C) for 50 minutes (Refrigerated Microcentrifuge, HERMLE Z 216MK; Hermle Labortechnik). After two centrifugations, approximately 1 mL of the supernatant was transferred to vials, filtered again, and immediately taken for analysis on the HPLC apparatus (SHIMADZU). In this device, we used the detector model SPD-20A VP, coupled to the UltraViolet (UV) detector, pump (LC-20AT), oven (CTO-20A), and autoinjector (SIL-20A HT). The wavelength was 210 nm, and the mobile phase elution system was isocratic.

The column conditions were as follows: Bio-Rad HPX-87H column, 300 mm × 4.6 mm; Guard column, Bio-Rad Cation H; and flow rate: 0.7 mL/min. The column temperature was 45°C, and the injection volume was 20 *μ*L. The results of acetic, propionic, and butyric acids were expressed in *μ*mol/gram of feces.

### 7.5. Statistical Analysis

Double data entering to avoid data entry mistakes and all statistical analyses of data were carried out using the Statistical Package for the Social Sciences (SPSS) for Windows, version 20.0 (IBM Corporation®, New York, United States).

The significance level for statistical tests was *α* = 5%. The Kolmogorov-Smirnov test and values for the statistics of skewness and kurtosis evidenced nonnormality in the measured variables. Therefore, results were presented as the medians and interquartile ranges (IQR). The Mann-Whitney and Kruskal-Wallis tests were used to test differences between two or more groups, respectively. The Bonferroni post hoc test was used to verify differences between pairs of groups.

The correlation between the continuous variables was evaluated through the significance of the Spearman coefficients (rs). Box plots were used to show possible associations of body composition measurements with abundance of bacterial phyla, SCFAs concentrations, and inflammatory markers.

**Figure 1:**
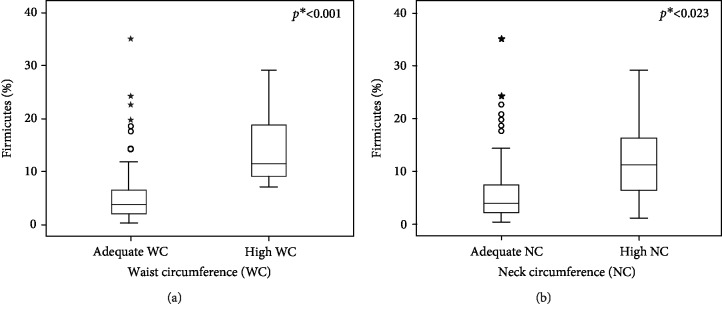
Firmicutes phyla abundance associated with waist and neck circumference in female adolescents (Viçosa, MG, Brazil, 2019). (a) Effect size: 0.826 (large Cohen's *d*). (b) Effect size:0.479 (medium Cohen's *d*). *N* = 96; ^∗^*p* value< 0.05.

**Figure 2:**
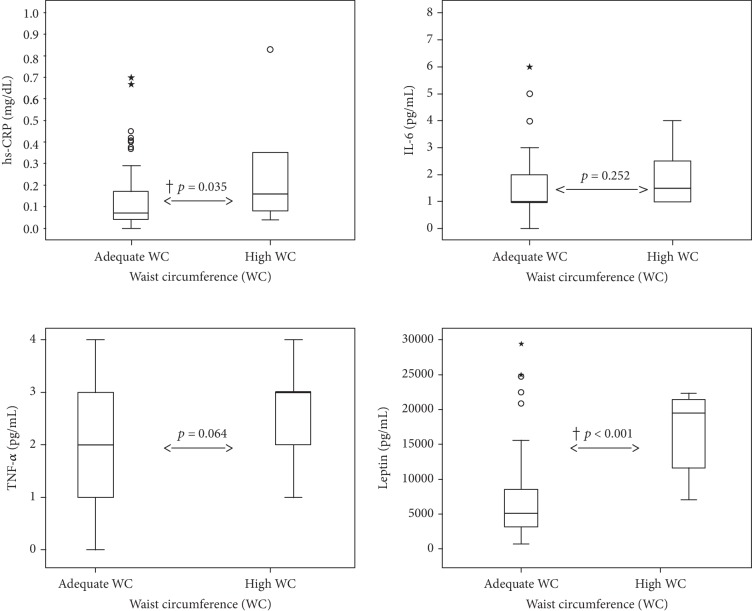
Association of inflammatory marker concentrations with waist circumference in female adolescents (Viçosa, MG, Brazil, 2019). Effect size of hs-CRP: 0.44 (medium Cohen's *d*). Effect size of IL-6: 0.215 (small Cohen's *d*). Effect size of TNF-*α*: 0.372 (medium Cohen's *d*). Effect size of leptin: 0.879 (large Cohen's *d*). ^†^*p* value of the Mann-Whitney test < 0.05. hs-CRP: high-sensitivity C-reactive protein; IL-6: interleukin-6; TNF-*α*: tumor necrosis factor-*α*.

Effect sizes were calculated for the differences between groups, such as those in Figures 1 and 2. For this, the calculator was used for the test statistics of the Wilcoxon signed-rank test, Mann-Whitney *U* test, or Kruskal-Wallis *H* test in order to calculate *η*^2^ [26, 27]. The effect sizes were classified according to the cutoff points suggested by Bakeman [28].

## 8. Results

The sample for this study consisted of 96 female adolescents. Nine girls were excluded from the study because they did not submit stool samples for the analysis of the intestinal microbiota phyla and SFCAs. The remaining participants underwent body composition evaluation, blood biochemical analysis, feces collection and analysis of bacterial phyla, and concentration of SCFA. G1 (eutrophic and adequate BF%) was composed of 31 participants (mean age 16.23 ± 0.76 years), G2 (eutrophic and high BF%) of 32 participants (mean age 16.53 ± 0.91 years), and G3 (overweight OR obesity and high BF%) of 33 participants (mean age 16.18 ± 1.26 years).

Female adolescents in G2 and G3 had higher waist circumference (WC) values (*p* < 0.001), waist to height ratio (WtHR) (*p* < 0.001), neck circumference (NC) (*p* < 0.001), android BF% (*p* < 0.001), gynoid BF% (*p* < 0.001), and leptin concentration (*p* < 0.001) (Table 2).

Insulin and HOMA-IR index concentrations were higher in G3 than in G1 (*p* < 0.001) and G2 (*p* < 0.001). Oil and fat consumption frequencies in groups G1, G2, and G3 were, respectively (5 (IQR 3-7), 6 (IQR 4-7), and 6.5 days/week (IQR 3.2-7)), without significant differences between groups (*p* = 0.844). The 80th percentile of fruit intake frequency was 6 days, which means 80% of adolescents reported not ingesting at least one fruit during all 7 days of the week.

The abundances of the Firmicutes, Bacteroidetes, and Proteobacteria phyla in addition to the concentrations of acetic, butyric, and propionic fatty acids were not different among the three groups of body composition (Table 3). The median of Firmicutes phylum and propionic acid in overweight or obese adolescents and those with high BF% was 5.5% (IQR: 2.5%-10.5%) and 6 *μ*mol/g (IQR: 5.0 *μ*mol/g-9.0 *μ*mol/g), respectively; groups G1 and G2 did not present significant differences.

The relationship between the abundance of bacterial phyla and SCFA concentrations with anthropometric measures, body fat, gynoid and android fat, food frequency, biochemical parameters, and inflammatory markers are presented in Table 4. The frequency of oil and fat consumption was negatively correlated with acetic acid (rs = ‐0.286, 95% CI: -0.0464 to -0.082, *p* = 0.005) and positively with butyric acid (rs = 0.324, 95% CI: 0.113 to 0.497, *p* = 0.002). The WC was related to the propionic acid concentration (rs = 0.205, 95% CI: 0.008 to 0.403, *p* = 0.042).

A previous analysis did not show an association between BMI and BF% with the intestinal microbiota phyla. On the other hand, the median amount of Firmicutes phylum verified in adolescents with high WC was 11.4% (IR 8.3%-19.8%), higher than the abundance found in those with adequate WC 3.9% (IR 2.2%-6.7%) (*p* < 0.001). Also, the girls with high NC had the median amount of Firmicutes higher than those with adequate NC, being 11.2% (IQR 6.0%-15.7%, *p* = 0.023) versus 4.1% (IQR 2.2%-7.5%) (*p* = 0.023), respectively (Figure 1). The effect size of the association between the abundance of Firmicutes and WC was 0.826 (a large Cohen's *d* value); for the association between the same phylum and NC, it was 0.479 (medium Cohen's *d*). These results confirm that NC and WC are anthropometric indicators associated with the abundance of the Firmicutes phylum.

There was no relation between the analyzed bacterial phyla or the concentration of SCFA and inflammatory markers. However, adolescents with high WC (>90thP) did show increased levels of inflammatory markers, namely, hs-CRP, TNF-*α*, and leptin, than those with adequate WC, as shown in Figure 2.

The effect sizes of the associations between inflammatory markers and WC, in terms of Cohen's *d* values, were considered large for leptin (0.879), medium for hs-CRP (0.44) and TNF-*α* (0.372), and small for IL-6 (0.215).

## 9. Discussion

The present study evaluated the abundance of Firmicutes, Bacteroidetes, and Proteobacteria, as well as the concentration of acetic, butyric, and propionic acids in the feces of female adolescents. Adolescents with high body fat presented higher values of anthropometric measurements (WC, NC, and WtHR), gynoid and android BF%, and higher concentrations of insulin, insulin resistance (IR), and leptin. Our results showed association of WC and frequency of oil and fat consumption with the abundance of Firmicutes and SCFA concentration. In addition, adolescents with high WC had higher concentrations of leptin and hs-CRP.

The girls classified as high BF% and overweight or obese had the highest values for WC, WtHR, and NC. The adolescents in group G2, who were eutrophic and had high BF%, also showed greater measures of central visceral adiposity markers. This highlights the importance of using other anthropometric indices in clinical evaluations for a more complete perception of the nutritional status and body composition of adolescents. Fedewa et al. [29] confirmed this necessity by showing in their study that, in addition to BMI, the WC was the anthropometric measure with the best association with body fat in young adults (21.8 ± 4.8 years old).

HOMA-IR, insulin, and leptin concentrations were higher in groups G2 and G3, both with high BF%, than G1, which was composed of eutrophic adolescents with adequate BF%. Also, in this case, body fat was more related to insulin and leptin concentrations than BMI, once again showing the importance of its evaluation in the early detection of risk factors for disease.

Leptin and insulin act to reduce food intake and increase energy expenditure via action on hypothalamic neurons; therefore, they are called “body adiposity signals” [30]. Adolescents with high WC had higher values of leptin and hs-CRP than those with adequate WC.

The deficiency of SCFA absorbed by the intestinal cells can decrease satiety [31] and lead to increased production of oxygen free radicals, which cause rupture of the enterocytes. This increases intestinal permeability by providing pathogen and lipopolysaccharide (LPS) entry into the intestinal lining [32]. The outer cell wall of gram-negative bacteria is composed of LPS molecules, which function as antigens and stimulate host immune response by activating Toll-like receptors [11].

In the present study, the similarity in abundance of GIT bacterial phyla and SCFA between G1, G2, and G3 can be explained by the fact that, in general, adolescents have protective factors that maintain the intestinal microbiota balance, such as better metabolism activity, higher energy expenditure, and low incidence of metabolic changes [33].

Groups G1, G2, and G3 presented similar food consumption frequencies. This may be because adolescents, in general, present a common dietary pattern with low consumption of fruits and vegetables and higher consumption of high-calorie, high-sugar, and preservative-rich foods [34, 35].

Unfortunately, the instrument used in this research to assess food frequency did not analyze the amount of fiber and other macro- and micronutrients ingested by girls. The greater consumption of hypercaloric foods and lower consumption of dietary fiber may, over time, influence the composition of the GIT bacterial phyla [4, 7, 36].

The possibility of preventing or treating obesity by modifying the intestinal microbiota has been stimulating a number of scientific studies in recent years [4, 8]. Up to the present moment, the specific intestinal microbiota profile of obese individuals has not been identified [3, 4]. The diversity of the human intestinal microbiota in adults and newborns (aged 0 to 2 years) has been examined in detail in another study [37]; however, little is known about the gut microbiota composition in adolescents, [38] a problem that our study sought to address.

Murugesan et al. [7] found no difference between Firmicutes, Bacteroidetes, Actinobacteria, and Proteobacteria in the GIT microbiota of 190 Mexican children and adolescents with ages 9 to 11, divided into eutrophic, overweight, and obesity groups. However, the obese children and adolescents displayed an abundance of the Lachnospiraceae (*p* = 0.018) family and the *Faecalibacterium* spp. (*p* = 0.042) and *Roseburia* spp. (*p* = 0.015). In another study by Bervoets et al. [8] (*N* = 26, BMI 28.7 ± 6.5), the Firmicutes/Bacteroidetes ratio was presented as higher in lean children (*n* = 27, BMI = 16.5 ± 2.1), and low relative proportions of *Bacteroides vulgatus*, high concentrations of *Lactobacillus* spp., and positive correlation with hs-CRP were observed in the microbiota of overweight children.

The highest amount of central visceral adiposity, evaluated by WC, showed correlation with the concentration of propionic acid (rs = 0.205, *p* = 0.042). Similar results have been found by Teixeira et al. [11] for adult Brazilian women. These authors observed that the concentrations of propionic and acetic acids correlated to BMI and body fat, confirming the association between the amount of SCFA found in adult women's feces and the increase in fat deposits [11].

The present study verified that female adolescents with high WC (*p* < 0.001) and high NC (*p* = 0.023) had higher Firmicutes abundance in relation to adolescents with adequate WC and NC (Figure 1). The results in the present study ensure that WC and NC are good predictors for cardiometabolic disease risk factors and, in addition, can present an association with intestinal microbiota concentrations in overweight female adolescents. Therefore, both WC and NC should be evaluated in clinical practice along with BMI, since these measurements have been shown to be useful tools to predict abdominal adiposity and to screen several chronic noncommunicable diseases during adolescence, such as hypertension, dyslipidemia, IR, and diabetes [39].

Currently, WC is a recommended central visceral adiposity marker for cardiometabolic risk screening and an alternative surrogate marker that has been recently proposed to better reflect the metabolic impact of central visceral adiposity accumulation per se [40]. NC has been used as an index for such an adverse risk profile. One study with Turkish adults confirmed that NC contributes to metabolic syndrome components and obstructive sleep apnea syndrome, being this association only with male adults [39]. Moreover, NC was found to have good discriminatory power with cutoff values of 36.55 cm for males and 34.05 cm for females, with superior sensitivity and specificity to predict overweight and obesity in comparison to direct BF% estimation [41].

Central visceral adiposity accumulation further enhances metabolic abnormalities but is not routinely measurable in clinical practice due to the invasiveness of the procedure as well as technical and financial issues [39]. Surrogate anthropometric markers for central visceral adiposity are, therefore, commonly used in addition to BMI for patient risk stratification, and WC and NC have been recommended for this purpose by recent clinical guidelines [42]. Moreover, according to our scientific evidence, NC and WC can be used as a predictor of gut microbiota abundance.

Some aspects have limited the results of the present study. For example, assessing only bacterial phyla may have contributed to the lack of difference in GIT bacterial phyla abundance between the groups of female adolescents classified by the BMI and the BF%. The phyla of the gut microbiota are composed of families, genera, species, and strains of bacteria that may differ between lean and obese individuals, although not much is known about that, especially among adolescents [3].

Despite some limitations, we believe this study captures important information from the joint evaluation of the intestinal microbiota and the concentrations of SCFA present in the feces of female adolescents, especially considering that few studies have investigated fecal bacteria phyla prevalence in adults [11] and even fewer in children and adolescents [7]. The association of the abundance of microbiota and its metabolites with the waist and neck circumferences emphasizes the importance of their evaluation and interpretation for the pediatric population. Therefore, we perceive the necessity of more epidemiological population-based studies to confirm the clinical relevance of NC and WC regarding metabolic syndrome components and gut microbiota composition.

## 10. Conclusions

Female adolescents with high BF% (G2 and G3) had higher NC, WC, WHtR, and percentages of gynoid and android fat, as well as higher insulin resistance index (HOMA-IR), insulin, and leptin concentrations. There was no difference in the abundance of the bacterial phyla between the body composition groups classified by BMI and BF%. However, girls with high WC and NC showed a greater abundance of Firmicutes. Also, WC was the marker for central visceral adiposity that showed association with inflammatory markers, such as hs-CRP and leptin concentrations.

We can state from the results found that NC and mainly WC should be incorporated in the nutritional status clinical evaluation of the adolescent population. Both anthropometric measurements are markers of central visceral adiposity that have been associated with cardiometabolic disease risk factors and also with the gut microbiota phyla abundance alongside their metabolites.

## Figures and Tables

**Table 1:** Specific indicator sequences for RT-qPCR analysis (Viçosa, MG, Brazil, 2019).

Groups	Primers (S and A)	Standard genomic DNA	References
Total bacteria	S—GCAGGCCTAACACATGCAAGTC	*Escherichia coli*	Castillo et al. [20].
	A—CTGCTGCCTCCCGTAGGAGT		

Bacteroidetes	S—CATGTGGTTTAATTCGATGAT	*Bacteroides vulgatus*	Guo et al. [21]
	A—AGCTGACGACAACCATGCAG		

Firmicutes	S—ATGTGGTTTAATTCGAAGCA	*Lactobacillus delbrueckii*	Guo et al. [21]
	A—AGCTGACGACAACCATGCAC		

Proteobacteria	S—CATGACGTTACCCGCAGAAGAAG	*Escherichia coli*	Friswell et al. [22]
	A—CTCTACGAGACTCAAGCTTGC		

RT-qPCR: real-time polymerase chain reaction; S: sense; A: antisense. All oligonucleotides were purchased from Alpha DNA and Molecular Diagnostics.

**Table 2:** Anthropometric measures, food frequency, and concentration of inflammatory markers in the three groups of female adolescents with respect to body composition (Viçosa, MG, Brazil, 2019).

*N* = 96 Variables	Group 1 (G1) (*n* = 31)	Group 2 (G2) (*n* = 32)	Group 3 (G3) (*n* = 33)				
	EUT and adequate BF%	EUT and high BF%	OW-OB and high BF%	G1, G2, & G3	G1 & G2	G1 & G3	G2 & G3
	Median (*P*_25_–*P*_75_)	Median (*P*_25_–*P*_75_)	Median (*P*_25_–*P*_75_)	*p* ^†^	*p* ^‡^	*p* ^‡^	*p* ^‡^
WC (cm)	62.3 (61.0-67.2)^†^	70.3 (68.1-75.3)	82.5 (78.7-88.2)	<0.001^∗^	<0.001^∗∗^	<0.001^∗∗^	<0.001^∗∗^
WtHR	0.4 (0.38-0.41)	0.43 (0.42-0.46)	0.50 (0.48-0.53)	<0.001^∗^	<0.001^∗∗^	<0.001^∗∗^	<0.001^∗∗^
NC (cm)	29.5 (28.0-30.0)	30.1 (29.2-31.0)	32.5 (31.0-34.0)	<0.001^∗^	0.001^∗∗^	<0.001^∗∗^	<0.001^∗∗^
Android fat (%)	12.6 (9.8-16.5)	23.0 (17.9-30.5)	37.3 (30.5-46.8)	<0.001^∗^	<0.001^∗∗^	<0.001^∗∗^	<0.001^∗∗^
Gynoid fat (%)	34.5 (30.6-36.7)	39.7 (37.9-46.9)	48.0 (45.5-54.1)	<0.001^∗^	<0.001^∗∗^	<0.001^∗∗^	<0.001^∗∗^
Fruits^a^	4.0 (1.5-6.5)	4.0 (2.0-5.0)	4.0 (1.2-6.7)	0.846	—	—	—
Vegetables^a^	6.0 (5.0-7.0)	6.0 (5.0-7.0)	7.0 (5.2-7.0)	0.846	—	—	—
Sugar and candies^a^	5.0 (4.5-7.0)	5.0 (4.0-7.0)	5.5 (3.0-7.0)	0.411	—	—	—
Oils and fat^a^	5.0 (3.0-7.0)	6.0 (4.0-7.0)	6.5 (3.2-7.0)	0.844	—	—	—
Total cholesterol (mg/dL)	142.0 (136.0-157.0)	141.0 (123.5-156.0)	150.0 (132.0-165.0)	0.301	—	—	—
HDL (mg/dL)	48.0 (41.0-54.0)	43.0 (38.2-57.5)	46.0 (38.0-54.0)	0.856	—	—	—
LDL (mg/dL)	81.0 (68.0-91.4)	76.6 (65.8-87.9)	88.0 (71.2-102.6)	0.195	—	—	—
Triglycerides (mg/dL)	68.0 (51.0-85.0)	61.0 (55.5-86.7)	68.0 (53.5-85.0)	0.986	—	—	—
Glucose (mg/dL)	84.0 (80.0-88.0)	86.0 (83.0-90.0)	86.0 (82.5-90.5)	0.276	—	—	—
Insulin (UI/mL)	5.9 (4.8-7.8)	5.7 (3.9-7.6)	9.5 (6.1-17.2)	<0.001	0.317	<0.001^∗∗^	<0.001^∗∗^
HOMA-IR (mg/dL)	1.24 (0.9-1.73)	1.2 (0.8-1.7)	2.04 (1.2-3.8)	<0.001^∗^	0.41	<0.001^∗∗^	<0.001^∗∗^
Hs-CRP (mg/dL)	0.06 (0.03-0.17)	0.08 (0.60-0.17)	0.08 (0.04-0.20)	0.33	—	—	—
TNF-*α* (pg/mL)	2.0 (1.0-3.0)	2.0 (1.0-3.0)	2.0 (1.0-3.0)	0.797	—	—	—
Interleukin-6 (pg/mL)	1.0 (1.0-2.0)	1.0 (1.0-2.0)	1.0 (1.0-2.0)	0.576	—	—	—
Leptin (pg/mL)	3226.0 (2632.8-4453.0)	5725.0 (3940.0-8422.5)	10377.5 (6645.7-20236.2)	<0.001^∗^	0.01^∗∗^	<0.001^∗∗^	<0.001^∗∗^

^†^Kruskal-Wallis test; ^‡^Dunns' post hoc test; ^∗^*p* < 0.05 in Kruskal-Wallis test; ^∗∗^*p* < 0.05 in Dunns' post hoc test. ^a^Average values of food frequency during one week. EUT: eutrophic; OW: overweight; OB: obesity; WC: waist circumference; WtHR: waist to height ratio; NC: neck circumference; HDL*:* high-density lipoprotein; LDL: low-density lipoprotein; HOMA-IR: Homeostasis Model Assessment-Insulin Resistance; Hs-CRP: high-sensitivity C-reactive protein; TNF-*α*: tumor necrosis factor-*α*.

**Table 3:** Phyla gut microbiota abundance and SCFA concentration of the three groups of body composition classification in female adolescents (Viçosa, MG, Brazil, 2019).

*N* = 96	Group 1 (G1) (*n* = 31)	Group 2 (G2) (*n* = 32)	Group 3 (G3) (*n* = 33)	
Bacteria phyla^∗^ and SCFA	Median (*P*_25_–P_75_)	Median (*P*_25_–*P*_75_)	Median (*P*_25_–*P*_75_)^a^	*p* ^†^
Firmicutes (%)	4.2 (2.3-8.5)	3.6 (1.9-7.3)	5.5 (2.5-10.5)	0.384
Bacteroidetes (%)	126.6 (94.3-154.7)	113.0 (77.5-136.7)	126.4 (91.9-141.5)	0.329
Proteobacteria (%)	3.2 (1.8-6.74)	2.0 (1.0-6.02)	2.5 (1.0-5.0)	0.23
Acetic acid (*μ*mol/g)	8.0 (7.0-10.0)	1.5 (6.0-9.7)	7.0 (6.5-10.0)	0.954
Propionic acid (*μ*mol/g)	5.0 (5.0-8.0)	5.0 (4.0-9.0)	6.0 (5.0-9.0)	0.395
Butyric acid (*μ*mol/g)	1.1 (0.6-1.9)	0.9 (0.6-1.9)	1.1 (0.6-1.6)	0.875

^∗^The abundance of phyla is expressed in percentages. ^†^Kruskal-Wallis test; SCFA: short-chain fatty acids; EUT: eutrophic; OW: overweight; OB: obesity; G1: eutrophic and adequate body fat percentage; G2: eutrophic and high body fat percentage; G3: overweight or obesity and high body fat percentage.

**Table 4:** Correlation between gut microbiota phyla, SCFA, body composition, food type frequency, and inflammatory marker concentrations in female adolescents (Viçosa, MG, Brazil, 2019)^a^.

*N* = 96 Variables	Firmicutes	Bacteroidetes	Proteobacteria	Acetic acid	Propionic acid	Butyric acid
BMI	0.088	-0.023	0.142	-0.076	0.0131	0.101
WC	0.099	0.004	-0.136	-0.038	**0.205** ^∗^^**c**^	0.048
WtHR	0.082	0.011	-0.171	-0.095	0.0149	0.047
NC	0.073	0.132	-0.159	-0.051	0.0156	0.057
BF%	0.086	-0.059	-0.011	-0.044	0.095	0.066
Android fat	0.146	-0.031	-0.181	-0.039	0.144	0.016
Gynoid fat	0.1	-0.083	-0.077	0.010	0.006	0.082
Fruits	0.112	0.014	0.002	-0.030	0.046	0.082
Vegetables	0.004	-0.020	0.034	-0.006	-0.005	-0.021
Oils and fats	0.037	-0.132	-0.022	-0.286^∗^^b^	-0.045	0.324^∗^^d^
Sugar and candies	0.122	-0.007	0.142	-0.036	0.137	0.129
Total cholesterol	-0.051	0.097	-0.113	0.011	0.018	-0.052
HDL	-0.003	-0.151	-0.05	-0.011	-0.129	0.022
LDL	-0.038	0.137	-0.158	0.019	0.07	-0.079
VLDL	0.119	0.133	0.019	-0.022	-0.002	0.025
Triglycerides	0.11	0.133	0.029	-0.022	-0.002	0.025
Glucose	-0.116	-0.088	-0.119	0.022	0.008	0.000
Insulin	0.118	-0.036	-0.095	-0.169	0.099	0.159
HOMA-IR	0.11	-0.031	-0.131	-0.149	0.011	0.136
Hs-CRP	0.063	-0.045	0.003	-0.165	-0.131	0.062
Interleukin-6	0.058	0.116	0.034	-0.117	0.000	0.146
TNF-*α*	-0.164	-0.025	-0.024	0.101	0.098	-0.102
Leptin	0.100	-0.125	-0.138	-0.149	0.141	0.129

BMI: body mass index; WC: waist circumference; WtHR: waist to height ratio; NC: neck circumference; BF%: body fat percentage; HDL: high-density lipoprotein; LDL: low-density lipoprotein; VLDL: very low-density lipoprotein; HOMA-IR: Homeostasis Model Assessment-Insulin Resistance; Hs-CRP: high-sensitivity C-reactive protein; TNF-*α*: tumor necrosis factor-*α*. ^a^Spearman's correlation coefficient values (rs); ^∗^*p* value of Spearman's correlation < 0.05. ^b^-0.286 (95% CI: -0.464 to -0.082); ^c^0.205 (95% CI: 0.008 to 0.403); ^d^0.324 (95% CI: 0.113 to 0.497).

## Data Availability

The data used to support the findings of this study was supplied by Data_VPMN_06-17-19.sav under a license and so cannot be made freely available. Requests for access to these data should be addressed to Dr. Valter P.N. Miranda (vpnmiranda@yahoo.com.br).

## Supplementary Materials

Supplementary Materials The supplemental material that was submitted was the database that was used in the data analysis.
